# Insights into the genomic evolution and the alkali tolerance mechanisms of *Agaricus sinodeliciosus* by comparative genomic and transcriptomic analyses

**DOI:** 10.1099/mgen.0.000928

**Published:** 2023-03-08

**Authors:** Zhi-Lin Ling, Bin Cao, Song-Nian Hu, Jia-Ning Geng, Fei Liu, Dong-Mei Liu, Rui-Lin Zhao

**Affiliations:** ^1^​ State Key Laboratory of Mycology, Institute of Microbiology, Chinese Academy of Sciences, Beijing 100101, PR China; ^2^​ College of Life Sciences, University of Chinese Academy of Sciences, Beijing 101408, PR China; ^3^​ Institue of Ecology, Chinese Research Academy of Environmental Sciences, Beijing 100012, PR China

**Keywords:** edible mushroom, saline-alkali environment, metabolic pathways

## Abstract

*Agaricus sinodeliciosus* is a rare wild edible mushroom from northwest China, and grows naturally in mild saline-alkali soil, which is also unusual in mushrooms. *A. sinodeliciosus* represents a potential model organism for explaining saline-alkali tolerance mechanisms and revealing related physiological processes in mushrooms. Here, we provide a high-quality genome of *A. sinodeliciosus*. Comparative genomic analyses reveal *A. sinodeliciosus* has numerous changes to its genome organization after a solitary evolutionary history under saline-alkali environments, such as gene family contraction, retrotransposon expansion and rapid evolution of adaptative genes. Our saline and alkali tolerance tests show that mycelium growth and fruit body formation of this species are effected by mild alkalinity. Transcriptomic analyses reveal that genes involved in carbon and nitrogen utilization, cell stability and fruit body formation of *A. sinodeliciosus* could be activated under mildly alkaline conditions. In particular, the ‘starch and sucrose metabolism’, ‘biosynthesis of amino acids’ and ‘phenylpropanoid biosynthesis’ pathways are important for mildly alkaline tolerance of *A. sinodeliciosus*. Like plants and arbuscular mycorrhizal fungi, in the rot fungus *A. sinodeliciosus*, the biosynthesis of intracellular small molecules could be enhanced to counter osmotic and oxidative stresses caused by mild alkalinity, and the biosynthesis of monolignol could be suppressed to increase cell wall infiltrates under mildly alkaline conditions. This research provides an understanding of the genomic evolution and mechanisms of *A. sinodeliciosus* in tolerance to saline-alkali environments. The *A. sinodeliciosus* genome constitutes a valuable resource for evolutionary and ecological studies of *Agaricus*.

## Data Summary

This Whole Genome Shotgun (WGS) project has been deposited at DDBJ/ENA/GenBank under accession JAHTMV000000000. The version described in this paper is version JAHTMV010000000. The WGS submission is associated with NCBI BioProject: PRJNA744025. The datasets of transcriptomic analyses used in this study are associated with NCBI BioProject: PRJNA772366. Alignments for phylogenetic analysis were submitted to TreeBASE (ID: 28495). The sequenced strain ZRL20160001-S65 was deposited at the China General Microbiological Culture Collection Center (CGMCC, Institute of Microbiology, Chinese Academy of Sciences, Beijing, China), with accession number CGMCC 14343.

**Impact Statement**

*Agaricus sinodeliciosus* as a rare, mild saline-alkali-tolerant mushroom, and may have great potential to provide a resource for meeting food needs and improving traits associated with mild saline-alkali tolerance in *Agaricus*. Little was known regarding the mechanisms of saline-alkali adaptation of rotting fungi. Here we sequence the genome of *A. sinodeliciosus*, and perform comparative genomic analysis to reveal the genome evolution of *A. sinodeliciosus* after a solitary evolutionary history under saline-alkali environments. Through saline and alkali tolerance tests, we illustrate the effect of mildly alkaline conditions for the growth and fruiting body formation of *A. sinodeliciosus*. Then combining transcriptomic analyses, we illustrate the mechanism of mildly alkaline tolerance of *A. sinodeliciosus*. Our research gives important clues for understanding of *A. sinodeliciosus* genome evolution and adaptive mechanisms under mild saline-alkali conditions.

## Introduction

Edible mushroom-forming fungi represent an important and sustainable food source, some of which have a delicious taste and favourable medicinal properties, and they also have immense economic and ecological significance [1, 2]. *Agaricus sinodeliciosus* (Agaricaceae, Agaricales, Agaricomycetes, Basidiomycota) is a wild edible mushroom which originates from northwest China [3]. The fruiting body of *A. sinodeliciosus* in the field is large, nutritious and flavourful, and widely consumed by locals [4]. Molecular phylogenetic research showed it has a close evolutionary relationship to the famous cultivated species *A. bisporus* [3].

In nature *A. sinodeliciosus*, which mainly inhabits mild saline-alkali soils, are semihypogeous in sandy soil along the reedy beaches of Bositeng Lake and Ebinur Lake in the Xinjiang Autonomous Region of China, and grassland of the Qaidam Basin in Qinghai Province of China [3–7]. Due to limited suitable habitat and indiscriminate mining, wild resources of *A. sinodeliciosus* are dwindling. Therefore, it is not only necessary to strengthen the protection of local wild resources, but also to develop sustainable ways to meet people’s food needs. Fortunately, experimental fruiting tests of *A. sinodeliciosus* showed that it can fructify under environmentally controlled conditions as well [4]. Therefore, *A. sinodeliciosus* was considered as another species for potential cultivation.

Organisms in nature often encounter soils with saline-alkali components, including NaCl which causes salt stress, and NaHCO_3_ and Na_2_CO_3_ which cause alkali stress. These kinds of soil can threaten their growth and development owing to ion toxicity, osmotic stress, metabolic disturbance and oxidative stress [8, 9]. For plants, alkali stress is more harmful than salt stress [10]. However, organisms also have developed a variety of mechanisms to protect against a saline-alkali environment. For example, plants have evolved a series of resistance pathways to maintain ionic homeostasis, adjust osmotic pressure, scavenge reactive oxygen species (ROS) and balance nutrition under saline-alkali environments [11].

Fungi also have evolved a series of mechanisms to adapt to changes in environmental conditions, including to a wide range of pH. Fungi have various pH regulatory systems that adapt gene expression responses to the ambient pH, including the Pal/Rim pathway, the calcium signalling pathway and the MDS3-dependent regulatory pathway [12–14], of which the Pal/Rim alkaline response pathway is specific to fungi [15, 16]. During the process of adapting to different pH environments, the physiological processes of fungi also have adjusted. For example, neutral and alkali pH conditions could significantly induce the expression of *GlPacC*, the homologous gene of the transcription factor PacC/Rim101 of the Pal/Rim pathway in *Ganoderma lucidum*, which also regulates various physiological processes, such as mycelium growth, fruiting body development, ion homeostasis and triterpene synthesis of secondary metabolites [17].

Studies regarding the tolerance of fungi in salt-alkali environments have mainly focused on arbuscular mycorrhizal fungi (AMF), which could improve the salt-alkali tolerance of their host plants [18–21]. AMF significantly improved tomato plant growth and reduced the harm from saline-alkali soil by optimizing the physiological processes of tomato plants, such as increasing the concentration of soluble solids, vitamin C (VC), soluble sugars, proline and lycopene in the fruit, increasing the absorption of nitrogen (N) and reducing the absorption of Na^+^ [18]. AMF colonization can improve growth and photosynthesis of *Leymus chinensis* seedlings under salt-alkali stress, by decreasing the Na^+^ content and increasing the K^+^ content to adjust their osmotic adjustment and ion balance [19, 20]. AMF colonization increased *Puccinellia tenuiflora* seedling biomass under alkali stress, through significantly increasing amino acid, organic acid, flavonoid and sterol contents to improve osmotic adjustment and maintain cell membrane stability [21].

Most mushrooms can grow and fruit well at pH around 6–7 [22]. The commercial compost used for the cultivation of *A. bisporus* has an approximately neutral pH of 7. However, *A. sinodeliciosus* as a highly prized edible mushroom is grown in mild saline-alkali soils. Therefore, we were interested in the genome evolution and physiological changes of *A. sinodeliciosus* under saline-alkali environments, and how such changes affect its nutrition and flavour.

In this study, we assembled the genome of *A. sinodeliciosus* using next generation sequencing (NGS) and single molecule real time (SMRT) sequencing, then integrated comparative genomic, salinity and alkalinity tolerance, transcriptomic analysis, gene expression and metabolic pathway analysis to evaluate the mechanisms and physiological processes of *A. sinodeliciosus* in tolerance to its environment, and finally inferred the possible changes of *A. sinodeliciosus* that may occur regarding its nutrition and flavour.

## Methods

### Genome sequencing

*A. sinodeliciosus* monokaryotic strain ZRL20160001-S65 isolated from single spore of the dikaryotic strain ZRL20160001 [23] was used for whole genome sequencing. Vegetative mycelia of ZRL20160001-S65 were cultivated in potato dextrose liquid medium at 25 °C for 2 weeks, then collected for DNA extraction. Genomic DNA was extracted using the cetyltrimethylammonium bromide (CTAB) method and purified by VAHTS DNA Clean Beads. Genome sequencing was conducted by the Illumina Hiseq x Ten and the PacBio RS II platform from Biomed. Two paired-end libraries (2×270 bp) and a PacBio library (20 kb) were constructed.

### Genome survey and assembly

In total, 10000 single-end reads of the Illumina NGS data were selected randomly to blast in the NT database for a contamination survey. Genome size, heterozygosity and repeat content were surveyed with a k-mer method using jellyfish [24] and GenomeScope [25]. Low-quality reads and libraries with short length reads <500 bp of the PacBio SMRT raw data were filtered using SMRT Link (Pacific Biosciences). Filtered SMRT subreads were assembled using WTDBG (https://github.com/ruanjue/wtdbg), and scaffolds were formed by SSPACE-LongRead [26]. A gap filling step was then carried out using PBjelly [27]. Finally, error correction was conducted by Pilon [28] combining NGS data. Genome-quality evaluation of the assembly was performed using three approaches: (1) paired-end reads were mapped to the assembled genome to calculate an error base percentage; (2) Benchmarking Universal Single-copy Orthologs (BUSCO) (v5.2.2, agaricales_odb10) [29] with default parameters was used to evaluate the quality of the assembled genome; and (3) assembled transcripts were mapped to the assembled genome.

### Genome annotation

Due to the relatively low conservation of interspecies repeats, a combination of *de novo* and homologue search strategies for identification and annotation of the repeats was used in the *A. sinodeliciosus* genome. RepeatModeler v.2.0.1 [30], which uses RECON [31] and RepeatScount [32], was applied with default settings to perform *de novo* identification and classification of repeat families from the assembled genome. Unknown classified repeats were further annotated through DeepTE [33]. MITE-Hunter [34] and LTR_retriever [35] were used to identify and classify the miniature inverted repeat transposable elements (MITEs) and long terminal repeat retroposons (LTR-RTs) sequences of the *A. sinodeliciosus* genome based on structural prediction. The *ab initio* predicted repeats were merged with the Repbase [36] database as the final repeats database, then RepeatMasker [37] was used to analyse and mask the repeats of *A. sinodeliciosus* based on the final repeats database. Intact LTR-RTs were identified and analysed using the LTR_retriever [35] pipeline, and the insertion time of different LTR-RTs types was subsequently obtained. For comparative purposes, repeats content of three closely related taxa (*A. bisporus* var*. bisporus*, *A. bisporus* var. *burnettii* and *Coprinopsis cinerea*) were also annotated.

Three kinds of strategies were used to predict protein-coding genes of *A. sinodeliciosus*, namely *ab initio* prediction, prediction based on homologous species, and validation based on transcripts and Unigene. Augustus [38], GlimmerHMM [39] and SNAP [40] were used for *ab initio* prediction, and GeMoMa [41] was used for prediction based on homologous taxa, *A. bisporus* var. *burnettii*, *A. bisporus* var. *bisporus* and *C. cinerea*. Then transcripts were aligned to the assembled genome using HISAT2 v.2.1.0 [42], and exons and introns were determined by StringTie v.1.3.3 [43] and TransDecoder v.5.0.0 (https://github.com/TransDecoder/TransDecoder). Finally, the predicted results were integrated using EvidenceModeler [44] to generate a reliable gene set.

The predicted genes were annotated by blast [45] searching against the NCBI NR database, KOG [46], GO [47], KEGG [48] and TrEMBL [49] databases. Secondary metabolite regions were identified by the anti-SMASH web-based analysis platform (version 5.2.0) [50]. The identification of cytochrome P450 (CYP) genes was performed following Chen *et al*. [51], using HMMER v3.2 (http://hmmer.org/) with hmmsearch of profile HMM Models derived from the Pfam seed alignment flatfile of PF00067, and positive CYPs were then annotated by blasting against the fungi CYP database (http://drnelson.uthsc.edu/CytochromeP450.html) [52]. The annotation of carbohydrate-active enzymes (CAZymes) and auxiliary activity enzymes (AAs) was based on HMM Models of the dbCAN2 database [53]. For comparative purposes, the contents of CYPs, CAZymes and AAs of 43 edible and medicinal fungi were also annotated.

Non-coding RNAs, such as microRNA, rRNA and tRNA, have been shown to have many functions. Different strategies are used to predict different non-coding RNAs based on structural characteristics. MicroRNA and rRNA were identified by a Blastn [45] search based on the Rfam [54] database, and tRNAs were identified using tRNAscan-SE [55].

Pseudogenes were predicted using BLAT [56] to search for potential genes from the gene-masked genome, and then GeneWise [57] was used to find immature stop codons and frameshift mutations in the potential genes.

### Phylogenetic tree construction and the variation of gene family analyses

Together with *A. sinodeliciosus*, another 43 sequenced edible and medicinal fungi (Table S1, available with the online version of this article) from the phyla Basidiomycota and Ascomycota were used in the phylogenetic analysis, and to study the evolutionary relationships among species. The genomic data of species were downloaded from the JGI database (https://genome.jgi.doe.gov/) and the NCBI database (https://www.ncbi.nlm.nih.gov/genome/). Orthologous gene families were selected and a phylogenetic tree was reconstructed following Prasanna *et al*. [58] with some modifications. An all-versus-all blast was used to cluster the predicted protein-coding gene of genomes using OrthoFinder [59] with DIAMOND [60] (e-value threshold 1e-5), which led to 303 single-copy orthologue gene families. The deduced protein sequences were aligned using muscle [61], and then the conserved regions were selected using Gblocks [62] with default parameters. A total of 282 single-copy orthologue gene families with conserved regions of ≥50 aa were concatenated. We partitioned the data set by gene, and the best-fit model for each partition was selected by ModelTest-NG [63] with gamma-distributed rate heterogeneity according to Akaike's information criterion (AIC). Maximum-likelihood (ML) phylogenetic inference including bootstrapping with 100 replicates was performed using RAxML-NG [64] with the best-fit partition model.

The divergence times of *Hymenochaetales*, *Agaricales* and *Trametes* [127–250, 105–210 and 45–90 million years ago (Mya), respectively] estimated by Varga *et al*. [65] were fixed in the molecule dating investigation. The divergence time of other nodes was estimated by r8s 1.81 [66], with parameters of the software set following Chen *et al*. [67]. CAFÉ 4.2.1 [68] was used to calculate the variation (expansion or contraction) of whole gene families, CAZymes and oxidoreductases in the 44 edible and medicinal fungi.

### Genome duplication and positive selection analyses among four closely related taxa

To reveal the genetic basis underpinning the *A. sinodeliciosus* phenotype (e.g. saline-alkali resistance), we investigated the evolution of gene families among four closely related taxa, *A. sinodeliciosus*, *A. bisporus* var. *bisporus*, *A. bisporus* var. *burnettii* and *C. cinerea*. The four closely related taxa have similar ecological niche (e.g. rotting straw), yet differ in terms of distribution, as *A. bisporus* var. *bisporus* and *C. cinerea* are widespread, while *A. sinodeliciosus* and *A. bisporus* var. *burnettii* have localized distributions. An all-versus-all blast was used to classify the predicted protein-coding gene of the four taxa using OrthoFinder [59] with DIAMOND [60] (e-value threshold 1e-5). Then the collinearity relationship at the nucleic acid level of genomes was obtained by MCScanX [69], and the circos diagram was finished by TBtools [70]. The duplicated genes of each taxon were classified by the duplicate_gene_classifier module in MCScanX. The Ka and Ks values of the syntenic orthologue gene pairs was calculated using the YN00 model in PAML [71], and genes with a ratio of Ka/Ks >1 were considered as positively selected genes.

### Tolerance tests of salinity and alkalinity in *A. sinodeliciosus*

Salinization and alkalinization of soil often occur together, characterized by salinity and increasing pH values, respectively. Additionally there are complex molecular mechanisms involved in the response and regulation to saline-alkali stress in organisms. To better understand how the two environmental factors impact the growth and development of *A. sinodeliciosus*, tolerance tests for salinity and alkalinity in *A. sinodeliciosus* were conducted separately. Commercial *A. bisporus* compost extract was used to prepare solid medium, which is more complex than potato dextrose agar (PDA) medium and could partly represents the natural environmental conditions.

In the salinity experiment, the vegetative mycelia of the wild strain ZRL20152597 were cultured at 25 °C in compost extract medium [72], with NaCl at 50, 100, 150 and 200 mmol l^−1^ NaCl, and a no-treatment control; growth diameters were measured every 3 days. In the alkalinity experiment, the pH of the sterile compost extract medium was determined using 0.1 mol l^−1^ NaOH, the vegetative mycelia of ZRL20152597 were cultured at 25 °C at pH 7.0, 7.5 and 8.0, and the growth diameters were measured every 3 days.

### Gene differential expression analyses

Mycelia of the wild strain ZRL20152597 grown at pH 7.0 and pH 8.0 for 15 days were collected separately for RNA-seq experiments, and each experiment was performed in biological triplicates. Total RNA was extracted from fresh mycelia using TRIzol reagent (Life Technologies) according to the manufacturer’s instructions. The degree of RNA degradation and contamination were analysed by agarose gel electrophoresis. RNA was quantified using a Nano Drop 2000 UV–vis spectrophotometer (Thermo Fisher Scientific) based on the absorbance at 260 and 280 nm, respectively. The quality of RNA also was measured via an Agilent 2100 bioanalyser (Agilent Technologies). RNA samples were subjected to sequencing via Majorbio.

RNA-seq was performed using an Illumina Hiseq. The raw data of each sample were filtered using Sickle [73] and SeqPrep [74]. Then, the clean reads of each sample were aligned separately to the assembled genome by Hisat2 [75] to obtain mapped reads. The read counts of mapped reads were calculated for each sample using RNA-seq by expectation maximization (RSEM), then transferred to fragments per kilobase of transcript per million fragments mapped (FPKM) to obtain standardized gene expression levels. The differentially expressed genes (DEGs) between two conditions were calculated by DESeq2 [76]. Finally, the DEGs were annotated by blasting against the KOG [46], GO [47], KEGG [48] and TrEMBL [49] functional databases.

### Gene co-expression network analyses

To examine the mechanisms of *A. sinodeliciosus* tolerance to mild alkalinity, we analysed the gene expression profiles of the mycelium of *A. sinodeliciosus* in different pH conditions to detect important functional elements with gene co-expression networks. Gene co-expression network analyses were conducted according to Schäpe *et al*. [77] with some modifications as follows. Spearman’s correlation coefficient [78] of DEGs was calculated using R. The calculated Spearman’s coefficients of DEGs were |≥0.5|, then |≥0.95| was taken as a threshold for co-expression. To visualize the gene co-expression network, the degree and betweenness of each node was calculated using the R package ‘igraph’ following Contreras-López *et al*. [79]. The visualization of gene networks and the creation of sub-networks were conducted using Cytoscape v.3.7.2 [80]. GO and KEGG enrichment of sub-networks were implemented using TBtools [70]. Hub genes in sub-networks were defined by node connectivity.

### Metabolic pathway reconstruction

We mapped annotated *A. sinodeliciosus* enzymes onto metabolic pathways using ‘KEGG Mapper – Reconstruct Pathway’ from the KEGG website.

## Results

### Genome assembly and annotation

A total of 2.22 Gb NGS raw data and 4.12 Gb SMRT sequencing raw data were produced. Through k-mer distribution analysis of the NGS data, the genome size of the monokaryotic strain ZRL20160001-S65 of *A. sinodeliciosus* was estimated to be ~32.03 Mb, of which the percentage of repeat sequences was ~24.65 %. The genome was *de novo* assembled based on SMRT subreads, and improved by a combination of NGS data. The final genome of 32.37 Mb was assembled, with scaffold N50 >1.46 Mb and contig N50 >1.02 Mb, respectively. Repeats represented 25.25 % of the *A. sinodeliciosus* genome, of which 23.33 % were transposon elements (TEs), and the most abundant TEs were LTR-RTs (17.08 %). Through a combination of different methods, 9 086 protein-coding genes were predicted to be present in the *A. sinodeliciosus* genome (Table 1). In addition, nearly 97 % of the transcripts of *A. sinodeliciosus* were aligned to the assembled genome. Additionally, 93.1 % of the agaricales_odb10 dataset of BUSCO could be completely detected in the assembled genome. In total, 98 % of the protein-coding genes of *A. sinodeliciosus* were annotated in multiple databases (Table 1). Seventeen secondary metabolite regions were detected using anti-SMASH (Tables 1 and S2), including eight gene clusters encoding key enzymes in terpene biosynthesis, six NRPS-like clusters, two type I polyketide synthases (T1pks), one gene cluster encoding key enzymes in indole biosynthesis, and one gene cluster encoding key enzymes in siderophore biosynthesis.

**Table 1.** Features of the *Agaricus sinodeliciosus* genome

General features	Count (%)
Number of scaffolds	58
Length of the genome assembly (Mb)	32.37
Scaffold N50 (kb)	1 460.65
Scaffold N90 (kb)	333.95
GC content	45.91 %
Length of repeats (Mb)	7.59 (23.36 %)
Number of protein-coding genes	9 086
Number of miRNA genes	0
Number of rRNA genes	14
Number of tRNA genes	123
Number of pseudogenes	403
**Predicted gene models**	
GO annotation genes	3 000 (33.02 %)
KEGG annotation genes	3 043 (33.49 %)
KOG annotation genes	4 609 (50.73 %)
Pfam annotation genes	6 065 (66.75 %)
Swissprot annotation genes	5 048 (55.56 %)
NR annotation genes	8 985 (98.89 %)
Transporter genes	78 (0.86 %)
Pathogen host interaction genes	299 (3.29 %)
Signal peptide	690 (7.59 %)
Transmembrane protein	1 658 (18.25 %)
Secreted protein	529 (5.82 %)
Anti-SMASH secondary metabolite regions	17
All annotation genes	8 986 (98 %)

GO, Gene Ontology; KEGG, Kyoto Encyclopedia of Genes and Genomes; KOG, Clusters of Orthologous Groups for Eukaryotic Complete Genomes; NR, Non-Redundant Protein Sequence Database.

### Phylogenetic analysis of *A. sinodeliciosus*

**Fig. 1.**
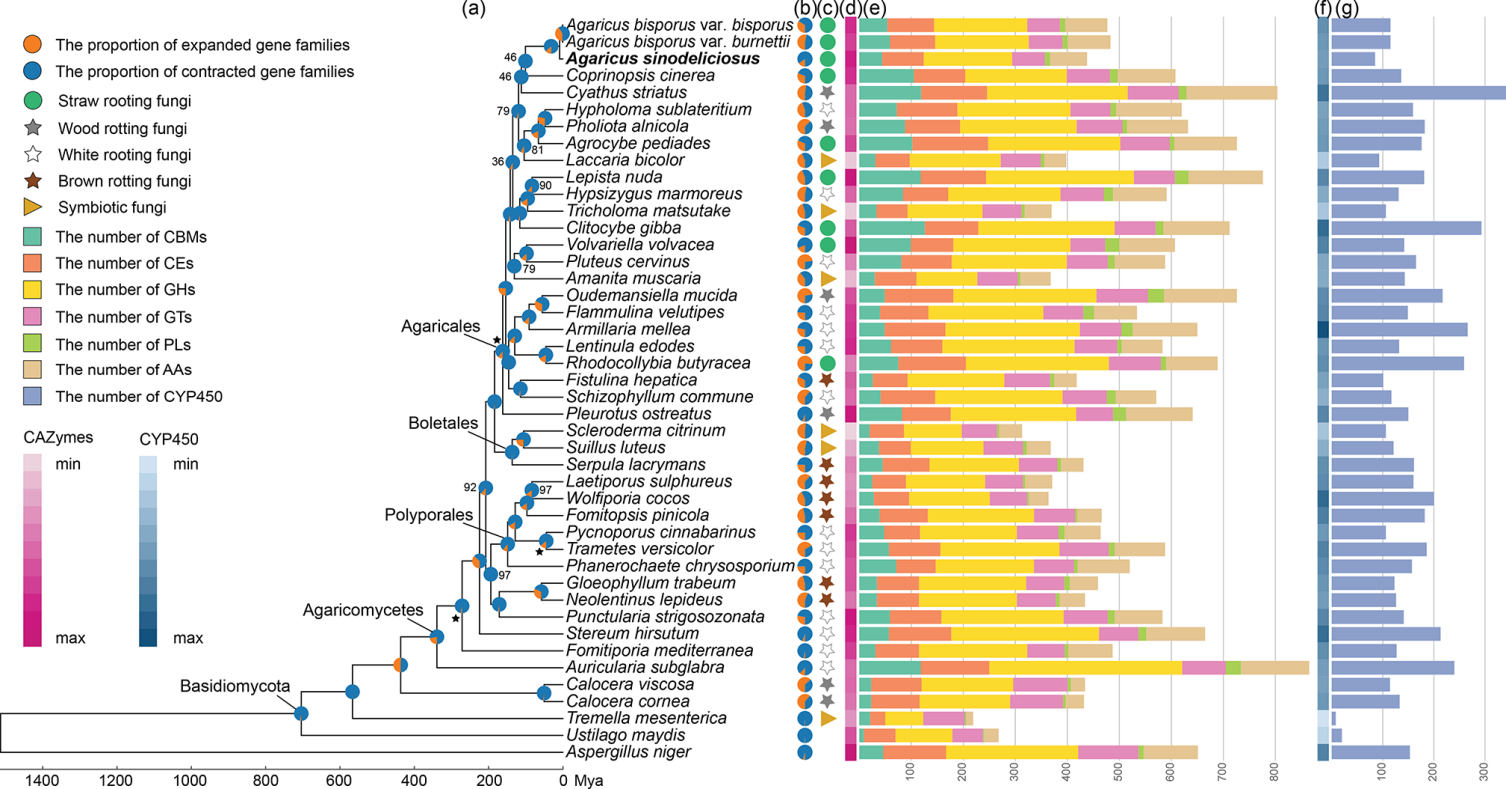
Phylogeny and gene family variation. (**a**) A phylogenetic tree of 44 edible and medicinal mushrooms. (**b)** Proportions of expanded gene families and contracted gene families in genomes. (**c)** Ecological niche of each species. (**d)** Heatmap of percentages of lignocellulolytic enzymes in genomes. (**e)** The content and gene numbers of lignocellulolytic enzymes in genomes. (**f)** Heatmap of percentages of CYP members in genomes. (**g)** The gene numbers of CYP members in genomes. Black stars indicate the used divergence time. Bootstrap support values are shown only for nodes with < 100 % support.

To illuminate the evolutionary history of *A. sinodeliciosus*, phylogenetic tree reconstruction and species divergence time estimation of 44 edible and medicinal fungi were conducted from 282 conserved single-copy orthologous proteins (Fig. 1a). Molecular dating revealed that *A. sinodeliciosus* has the closest evolutionary affinity with *A. bisporus*, which is consistent with the published results [3, 81], and we estimated that their divergence time was 8.89 Mya.

### Gene family evolution analysis revealed extensive contraction of gene families in *A. sinodeliciosus*

The predicted proteome of *A. sinodeliciosus* was compared with 43 sequenced edible and medicinal fungi. Gene family analysis showed that a total of 33 199 gene families were constructed. The expansion and contraction of gene families in all 44 fungi were examined using CAFÉ, and 174/968 gene families were found to have undergone expansion/contraction in *A. sinodeliciosus* (Fig. 1b, Table S3). GO enrichment analysis of the expanded gene families found them to be enriched in the cell wall and the extracellular region, as well as involved in oxidoreductase activity and ion binding. KEGG enrichment analysis of the expanded gene families found them to be enriched in the categories of metabolism, such as methane, energy and amino acid metabolism.

In addition, to reveal the genetic basis for lignocellulosic degradation of *A. sinodeliciosus*, we investigated the evolution of lignocellulolytic enzymes among 44 fungi, including CAZymes, AAs and CYPs. The CYP superfamily is also involved in the biosynthesis of secondary metabolites. In *A. sinodeliciosus*, 367 CAZymes, 71 AAs and 85 CYPs were identified. The average gene number of CAZymes, AAs and CYPs in fungi varies typically according to different ecological niches; for example, straw rotting fungi have the highest average number of CAZymes, AAs and CYPs, followed by white rotting fungi, brown rotting fungi and symbiotic fungi (Tables 2 and S4). *A. sinodeliciosus* has the lowest gene number of CAZymes and AAs compared to the genomes of other straw rotting fungi (438 genes compared to 483–776 genes) (Fig. 1e, Table S4), while it has a relatively high percentage of CAZymes and AAs in the genome (4.82 % compared to 3.01 –5.48 %) (Fig. 1d, Table S4). Except *Ustilago maydis* (20 CYP genes) and *Tremella mesenterica* (eight CYP genes), which have a small number of CYPs, *A. sinodeliciosus* has the lowest gene number of CYPs compared to the genomes of the other 41 selected fungi (85 compared to 93–341 CYP genes) (Fig. 1g, Table S4), and it has a relatively low percentage of CYPs in the genome (0.94 % compared to 0.40–1.84 %) (Fig. 1f, Table S4).

**Table 2.** The average gene number and standard deviation of CAZymes, AAs and CYPs in different ecological niche fungi included in this study

Family	White rotting fungi	Brown rotting fungi	Straw rotting fungi	Symbiotic fungi
CAZymes	495±79	374±35	508±95	297±48
AAs	98±19	47±5	105±22	43±14
CYPs	164±45	150±33	167±65	96±42

AAs, auxiliary activities; CAZymes, carbohydrate-active enzymes; CYPs, cytochromes P450 genes.

In total, 24 CAZyme and four AA families were found to have undergone contraction in *A. sinodeliciosus*; these gene families are involved in depolymerization of lignin (AA3, AA5, AA7), cellulose (CBM1, GH5, AA9), hemicellulose (CE7, CE16, GH31, GH43, GH51, GH78) and pectin (CE12, CE8, GH28, GH78). The only expanded CAZyme family in *A. sinodeliciosus* is CE1, which involved in decoupling lignin from polysaccharides in lignocellulose. Ten CYP families were found to have undergone contraction in *A. sinodeliciosus*; these gene families are involved in primary metabolism and secondary metabolism, including xenobiotic degradation (CYP63), reduction of nitrite (CYP55), degradation of various fatty acids and hydrocarbons (CYP530), sterigmatocystin biosynthesis (CYP62) and triterpenoid biosynthesis (CYP512).

### Decreasing gene duplications may result in extensive contraction of gene families in *A. sinodeliciosus*

**Fig. 2.**
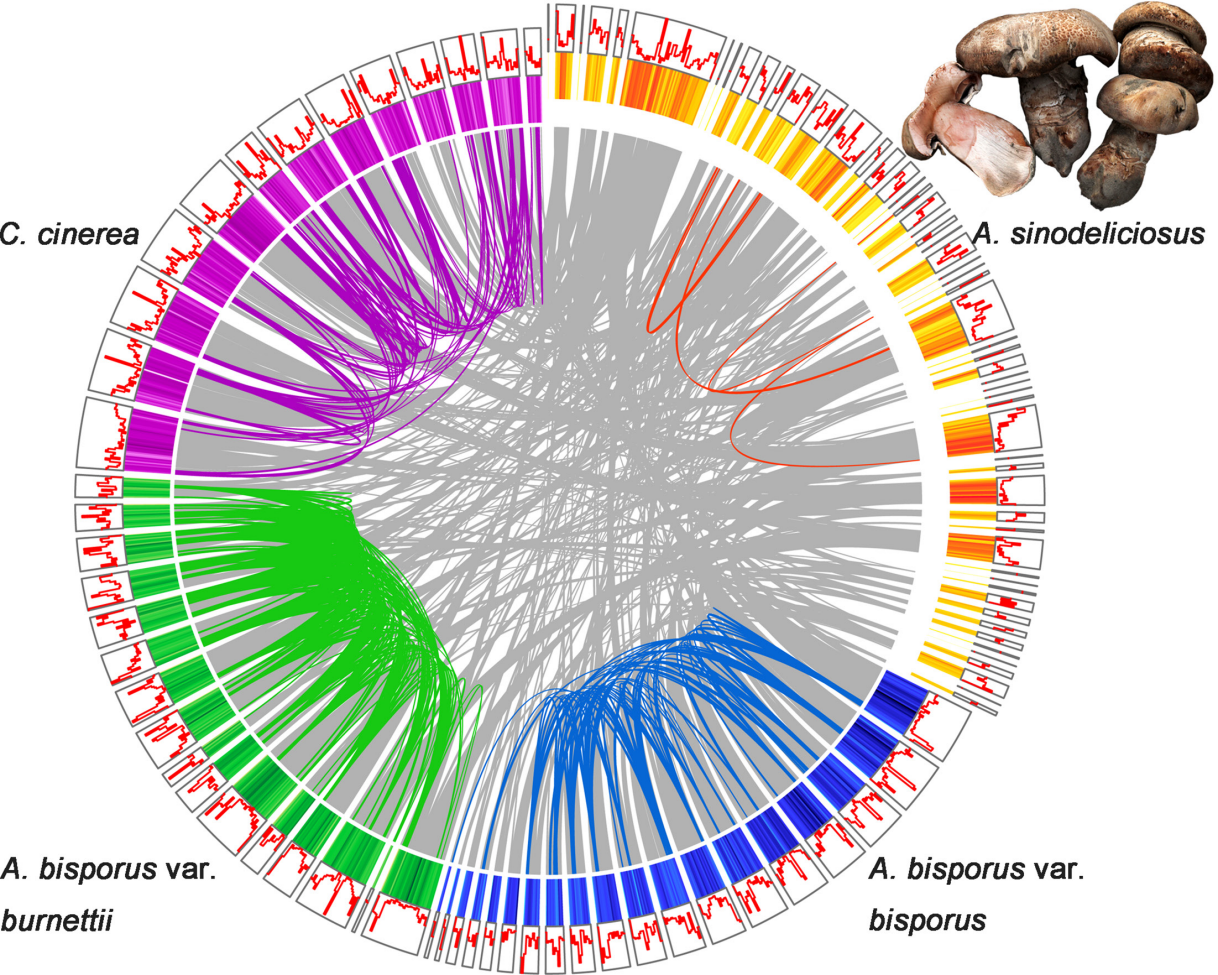
Genomic architecture of the *A. sinodeliciosus* genome and three closely related taxa. Scaffolds including more than 50 genes are shown in the circos diagram. Proteins sharing similarities between *A. sinodeliciosus* and other taxa are linked by grey lines. Intraspecific synteny is linked by orange, blue, green and purple lines, respectively. The orange, blue, green and purple circles represent protein-coding gene density. The red circle represents repetitive sequence density.

Compared to *A. bisporus* var. *bisporus* (10 450 protein-coding genes), *A. bisporus* var. *burnettii* (11 763) and *C. cinerea* (13 356), *A. sinodeliciosus* has a relatively smaller proteome size (9086). Self-comparison of each taxon's coding genes was performed to detect gene duplications. Out results supported extensive gene duplications having occurred in *A. bisporus* var. *burnettii*, in which duplicated genes covered 61.23 % of protein-coding genes, following by *C. cinerea* (58.25 %) and *A. bisporus* var. *bisporus* (57.00 %), while fewer gene duplications were observed in *A. sinodeliciosus* (52.15 %) (Table S5). Notably, the number of whole-genome duplications (WGD) in *A. bisporus* var. *burnettii* was higher than those found for *C. cinerea*, *A. bisporus* var. *bisporus* and *A. sinodeliciosus* (Fig. 2, Table S5).

### TEs were significantly expanded in the genome of *A. sinodeliciosus*

**Fig. 3.**
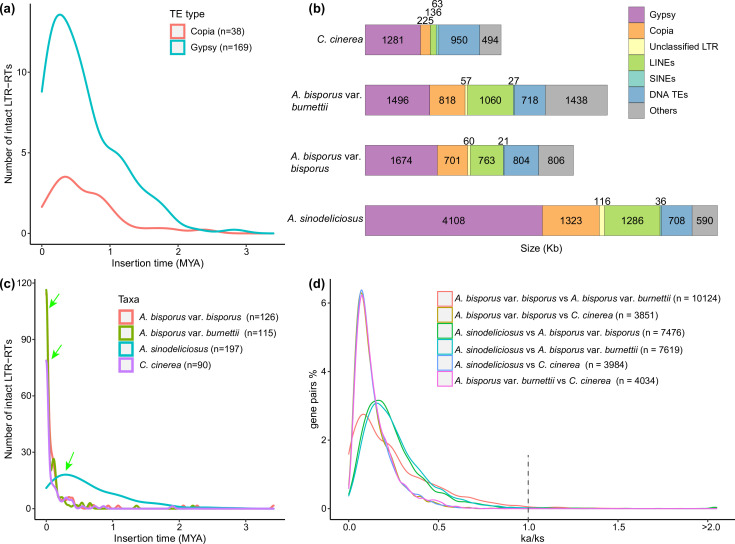
Analysis of TEs and positively selected genes in the *A. sinodeliciosus* genome and three closely related taxa. (**a)** Insertion bursts of *Gypsy* and *Copia* elements in *A. sinodeliciosus*. The numbers of intact elements used in this analysis are provided in parentheses. (**b)** Comparison of TE families in the four taxa. (**c)** Comparison of temporal patterns of intact LTR-RT insertion bursts in the four taxa. Green arrows indicate the peaks of intact LTR-RT insertion times. The number of intact LTR-RTs used for each taxon is given in parentheses. (**d)** Frequency distributions of Ka/Ks between homologous gene pairs of the four taxa. The number of homologous gene pairs between two taxa is given in parentheses.

The TE content in *A. sinodeliciosus* (23.33% of the 32.37 Mb genome) was clearly higher than identified in *A. bisporus* var. *bisporus* (15.53 % of the 30.78 Mb genome), *A. bisporus* var. *burnettii* (13.60 % of the 30.70 Mb genome) and *C. cinerea* (7.29 % of the 36.29 Mb genome) (Fig. 3b). Cross-genome comparisons showed that LTR-RTs contributed the most to the TE expansion of *A. sinodeliciosus* (Fig. 3b, c). *A. sinodeliciosus* had 5.55 Mb of LTR-RTs, including *Gypsy*, *Copia* retroelements and unclassified LTR elements, which were the dominant TEs and occupied 17.08 % of the genome (Fig. 3b). By contrast, LTR-RTs represented 8.89, 7.72 and 4.12 % of the genomes of *A. bisporus* var. *bisporus*, *A. bisporus* var. *burnettii* and *C. cinerea* (Fig. 3b).

A continuous insertion of intact LTR-RTs in *A. sinodeliciosus* since the Quaternary ice age 2 Mya was observed, and it had a distinct unimodal distribution (Fig. 3a, c). By contrast, there were latest insertion times nearly 0 Mya of intact LTR-RTs in *C. cinerea*, *A. bisporus* var. *bisporus* and *A. bisporus var*. *burnettii* (Fig. 3c). Therefore, *A. sinodeliciosus* had accumulated the most intact LTR-RTs among the four related taxa (Fig. 3a‒ c). At the superfamily level, *Gypsy* retrotransposons in *A. sinodeliciosus* had a recent burst around 0.3 Mya, and *Copia* elements in *A. sinodeliciosus* had peaks of amplification appearing around 0.4 Mya (Fig. 3a). Therefore, recent large-scale bursts of LTR-RTs at approximately 0.3–0.4 Mya contributed to the TE expansion of *A. sinodeliciosus*.

### Genes positively selected in *A. sinodeliciosus*

Positively selected genes in which Ka/Ks>1 were selected in *A. sinodeliciosus* versus *A. bisporus* var. *bisporus* (69 genes), *A. sinodeliciosus* versus *A. bisporus* var. *burnettii* (25 genes), and *A. bisporus* var. *bisporus* versus *A. bisporus* var. *burnettii* (139 genes), respectively (Table S6, Fig. 3d). Annotated, positively selected genes of *A. sinodeliciosus* included transcription factors such as zinc finger, regulatory protein cys-3, rpb7-binding protein seb1, and mushroom formation-related genes, for instance hydrophobin-3 and ricin-type beta-trefoil lectin.

### Salt and mild alkaline tolerance tests of *A. sinodeliciosus*

**Fig. 4.**
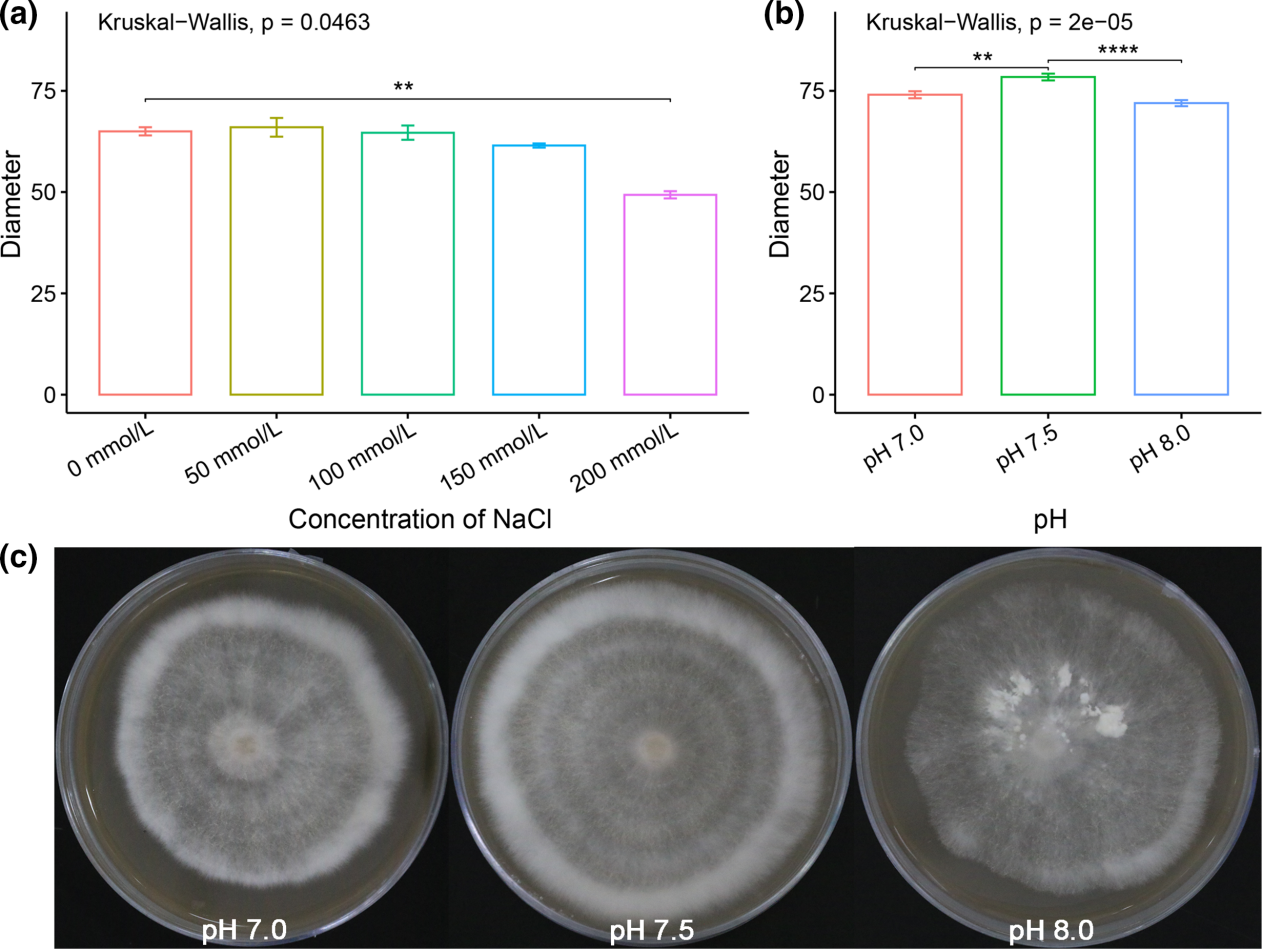
Tolerance tests of salinity and alkalinity in *A. sinodeliciosus*. (**a)** Mycelium growth diameters of *A. sinodeliciosus* at different concentrations of NaCl for 15 days; means with standard deviation are presented on the bar graph. (**b)** Mycelium growth diameters of *A. sinodeliciosus* at different pH conditions for 15 days; means with standard deviation are presented on the bar graph. (**c)** Hyphae of *A. sinodeliciosus* at different pH conditions. *P*<0.05 (*), *P*<0.01 (**), *P*<0.001 (***), *P*<0.0001 (****).

In the salinity experiment, *A. sinodeliciosus* presented no sensitivity to low and to moderate concentrations of salt (0–150 mmol l^−1^ NaCl) in compost extract medium. Limited growth was observed in a high concentration of salt (0 vs. 200 mmol l^−1^ NaCl, *P*=0.0013) (Fig. 4a). In the alkalinity experiments, *A. sinodeliciosus* grew more effectively at pH 7.5 vs. pH 7.0 (*P*=0.0013) and pH 7.5 vs. pH 8.0 (*P*<0.0001) in compost extract medium, and the growth rates showed no difference at pH 7.0 vs. pH 8.0 (Fig. 4b, c), which suggests that *A. sinodeliciosus* could grow well under mildly alkaline conditions at pH 8.0. Furthermore, the mycelium of *A. sinodeliciosus* starts to present hyphal knots at pH 8.0 (Fig. 4c).

### Gene expression of *A. sinodeliciosus* under different pH conditions and functional analysis of DEGs

Based on the salt and mild alkaline tolerance tests, to further investigate the related genetic mechanisms, transcriptomes of mycelia of *A. sinodeliciosus* grown under neutral (pH 7.0) and mild alkaline (pH 8.0) conditions were generated by RNA-seq using Illumina Hiseq. Replications with gene expression correlation coefficients >0.92 were selected for analysis. DEGs were selected with fold change ≥2 and *p*-adjust <0.05 as cutoffs, and 421 genes were up-regulated and 233 genes were down-regulated in the mycelia of *A. sinodeliciosus* cultured in pH 8.0 medium versus pH 7.0 medium (Table S7). The expression of seven important gene categories in DEGs were strongly associated with changes of environmental pH; most CAZymes involved in lignocellulose degradation, lipases related to lipid catabolism, peptidases related to nitrogen source utilization, transmembrane transporters participating in transmembrane transportation and genes related to mushroom formation were up-regulated at pH 8.0, while protein kinases which are crucial components of diverse signalling pathways in response to environmental stresses were all up-regulated at pH 7.0, while a variety of transcription factors were differentially expressed at pH 8.0 and pH 7.0 (Table S7, Fig. S1). Of these, 43.75 % of CAZymes, 20.00 % of lipases and 34.78 % of peptidases are secreted proteins (Table S7).

In GO enrichment analysis, the up-regulated DEGs at pH 8.0 were enriched in categories including extracellular region, hydrolase activity and carbohydrate metabolism, and the up-regulated DEGs at pH 7.0 were enriched in categories including oxidoreductase and antioxidant activity. In KEGG pathway enrichment analysis, the up-regulated DEGs at pH 8.0 were enriched for sugar metabolism (such as starch and sucrose), amino acid metabolism (including tyrosine, tryptophan, cysteine and methionine), lipid metabolism (such as glycosphingolipid) and secondary metabolism (such as folate, ubiquinone and other terpenoids–quinone biosynthesis) pathways; and the up-regulated DEGs at pH 7.0 were enriched for amino acid metabolism (including tryptophan, arginine and proline), lipid metabolism (such as ether lipid and glycosphingolipid), nitrogen metabolism and secondary metabolism (including glutathione, dibasic acid, taurine, hypotaurine, ubiquinone and other terpenoids–quinone) pathways.

### Gene co-expression network analysis

**Fig. 5.**
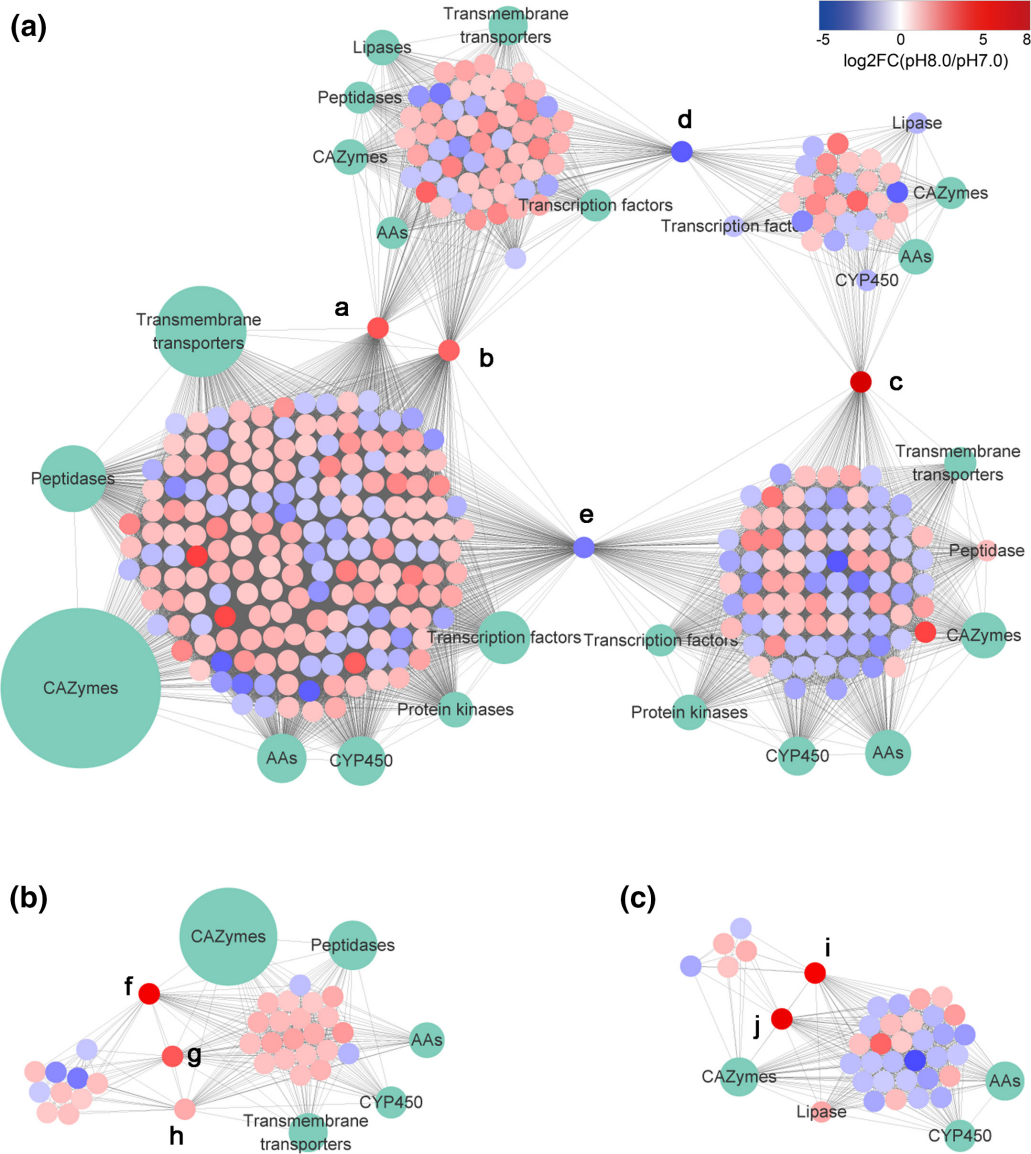
Gene co-expression network of differentially expressed genes (DEGs). (**a)** Sub-network 1. (**b) **Sub-network 2. (**c)** Sub-network 3. Letters a to j are hub genes in sub-networks. Blue and red circles indicate the expression pattern of DEGs (including up-regulated, down-regulated and fold change). Size of blue and red circle indicates one gene. Green circles indicate gene groups which perform the same function.

Co-expression networks were created to associate genes that are involved in similar biological processes, and provide hypotheses about key genes and implicated biological functions. Gene expression data of DEGs were used to create gene co-expression networks based on Spearman’s correlation coefficient. Three sub-networks (1–3) containing 48–502 nodes each were generated, and the functions of these sub-networks were enriched through GO and KEGG enrichment analysis (Fig. 5, Table S7).

In the sub-network 1 (Fig. 5a), the function of up-regulated DEGs at pH 8.0 were enriched in structural constituents of the cell wall, membrane, transmembrane transport and localization; while the function of up-regulated DEGs at pH 7.0 were enriched in ion binding, heterocyclic compound binding, protein kinase activity, intracellular, macromolecule modification, energy metabolism, etc. In sub-network 2 (Fig. 5b), the function of up-regulated DEGs at pH 8.0 were enriched in hydrolase activity, peptidase activity, extracellular region, carbohydrate metabolism, proteolysis, etc. Carbohydrate metabolism included cellular polysaccharide, glucan, starch, sucrose, oligosaccharide and disaccharide metabolic processes. Regarding the functions of genes in sub-network 3 (Fig. 5c), up-regulated DEGs at pH 8.0 were enriched in carbohydrate metabolism; up-regulated DEGs at pH 7.0 were enriched in oxidoreductase activity.

Furthermore, a total of 10 hub genes with greatest node relevance in each sub-network were identified. Five hub genes were identified in sub-network 1, including nitroreductase (Fig. 5a), voltage-gated potassium channel complex (Fig. 5b), IMP cyclohydrolase (purH) (Fig. 5c), serine/threonine protein kinase (Fig. 5d) and antibiotic biosynthesis monooxygenase (ABM) (Fig. 5e). Three hub genes were identified in sub-network 2, including polyketide cyclase/dehydrase and lipid transport gene (Fig. 5f), glycosyl hydrolase family 17 (GH17) (Fig. 5g) and DNA-directed RNA polymerase I subunit (RPA43) (Fig. 5h). Two hub genes were identified in sub-network 3, including rare lipoprotein A (RlpA)-like double-psi beta-barrel (DPBB) (Fig. 5i) and chitinase (GH18) (Fig. 5j).

### Key metabolic pathways may be related to mild alkali tolerance of *A. sinodeliciosus*

**Fig. 6.**
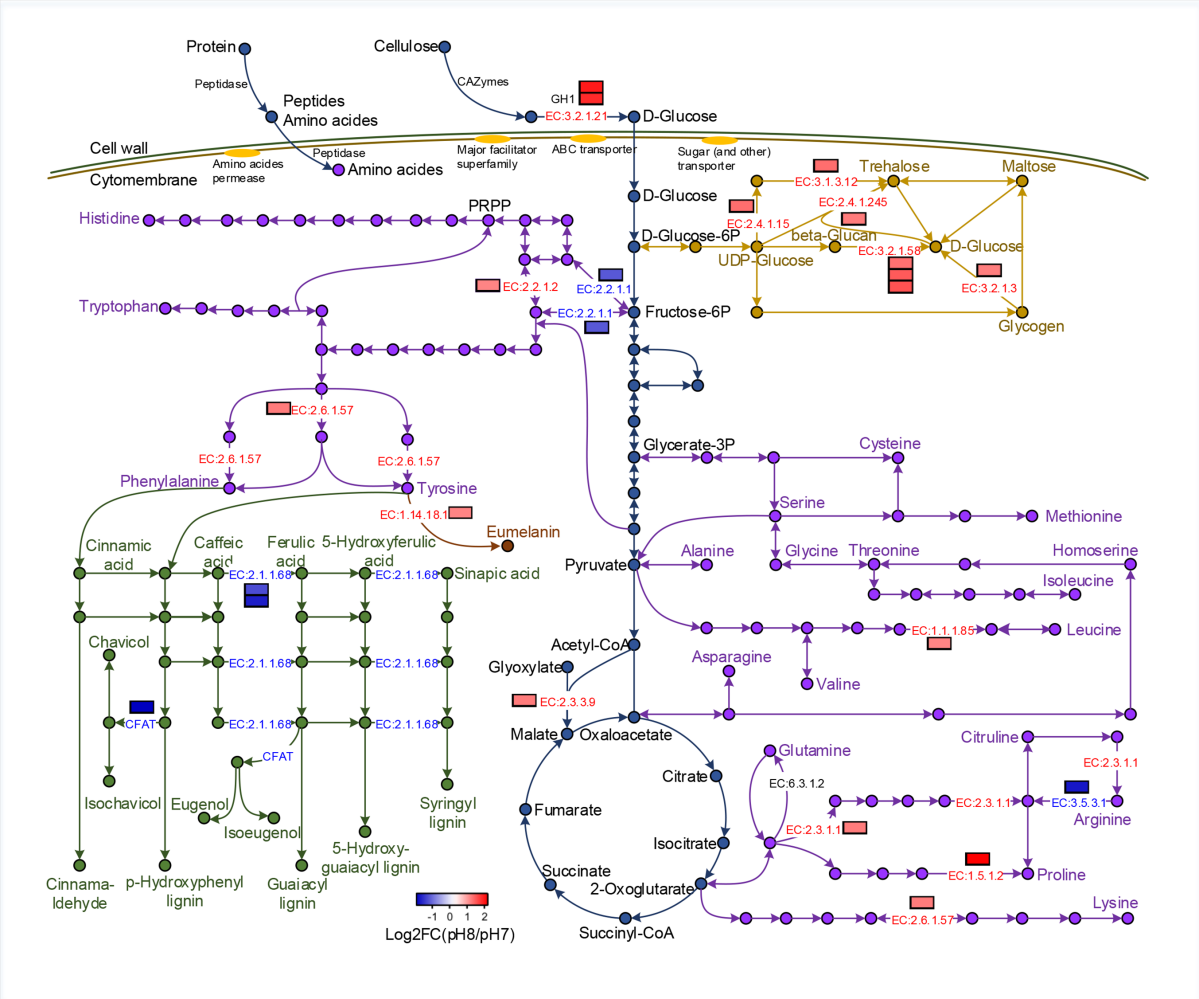
The important pathways of *A. sinodeliciosus* involved in tolerance to mild alkali conditions and the differential expression of enzymes. The ‘starch and sucrose metabolism’, ‘biosynthesis of amino acids’ and ‘phenylpropanoid biosynthesis’ pathways are represented by orange, purple and green, respectively. The arrows represent the enzymes that were identified in *A. sinodeliciosus*, and each dot represents the metabolite catalysed by the enzyme. Differentially expressed enzymes are represented as bar charts, and EC numbers of enzymes are embedded in the arrows.

To further explore the mechanisms of *A. sinodeliciosus* in tolerance to a mild alkali environment, the differentially expressed metabolites involved in three metabolic pathways were measured and summarized, including those involved in ‘starch and sucrose metabolism’, ‘biosynthesis of amino acids’ and ‘phenylpropanoid biosynthesis’ (see simplified metabolic map in Fig. 6).

For the reconstructed ‘starch and sucrose metabolism’ pathway in *A. sinodeliciosus*, genes related to glucan 1,3-beta-glucosidase [EC:3.2.1.58] and glucoamylase [EC:3.2.1.3], which are involved in d-glucose synthesis, were up-regulated at pH 8.0. Expression of the trehalose 6-phosphate synthase (TPS) gene [EC:2.4.1.15] and trehalose 6-phosphate phosphatase (TPP) gene [EC:3.1.3.12], which are involved in trehalose synthesis from UDP-glucose, were also up-regulated at pH 8.0. Gene expression of trehalose synthase (treT) [EC:2.4.1.245], which is involved in trehalose synthesis from UDP-glucose and d-glucose, were up-regulated at pH 8.0.

*A. sinodeliciosus* has 20 amino acid biosynthetic pathways, of which lysine synthesis through the a-aminoadipate (AAA) pathway, and the AAA pathway are present only in euglenoids and higher fungi [82]. A gene related to aromatic-amino-acid transaminase (tyrB) [EC:2.6.1.57] involving phenylalanine and tyrosine biosynthesis was up-regulated at pH 8.0, and gene expression of tyrosinase (TYR) [EC:1.14.18.1], which is the rate-limiting enzyme for melanin synthesis, also known as polyphenol oxidase (PPO), was also up-regulated at pH 8.0. Genes related to 3-isopropylmalate dehydrogenase (leuB) [EC:1.1.1.85] involved in leucine biosynthesis, *N*-acetylglutamate synthase (NAGS) [EC:2.3.1.1] involved in arginine biosynthesis, pyrroline-5-carboxylate reductase (proC) [EC:1.5.1.2] involved in proline biosynthesis, and aromatic amino acid aminotransferase I (ARO8) [EC:2.6.1.57] involved in lysine biosynthesis were additionally up-regulated at pH 8.0. The glutamine synthetase (glnA) [EC:6.3.1.2] gene involved in glutamine biosynthesis was highly expressed in *A. sinodeliciosus* (pH 7.0 FPKM 1959.63; pH 8.0 FPKM 1780.53).

In the reconstructed ‘phenylpropanoid biosynthesis’ pathway in *A. sinodeliciosus*, two genes related to caffeic acid 3-*O*-methyltransferase (COMT) [EC:2.1.1.68], which is a key enzyme for the synthesis of phenolic acids (ferulic acid, 5-hydroxyferrulic acid, sinapic acid) and monolignol (guaiacyl lignin, 5-hydroxy-guaiacyl lignin, syringyl lignin) derived from caffeic acid, were down-regulated at pH 8.0. A gene related to coniferyl alcohol acyltransferase (CFAT) [EC:2.3.1.-], which is involved in chavicol and isochavicol synthesized from 4-coumaryl alcohol, and eugenol and isoeugenol synthesized from coniferyl alcohol, was also down-regulated in response to pH 8.0.

## Discussion

*A. sinodeliciosus* is a mild saline-alkali-tolerant mushroom in nature, closest to *A. bisporus* in molecular phylogeny with an estimated divergence time of 8.89 Mya. A 32.37 Mb high-quality genome was assembled from a monokaryotic strain of *A. sinodeliciosus*, using long reads (PacBio) and rectified using accurate reads (Illumina). The genome evolution of *A. sinodeliciosus* was explored based on comparative genomic analyses of 44 edible and medicinal mushrooms, and of four closely related taxa.

In order to identify how environmental factors have impacted the growth and development of *A. sinodeliciosus*, we first performed saline and alkali tolerance tests. Although the composition of saline-alkali soil is complex, our study showed that pH is a key factor affecting the growth and development of *A. sinodeliciosus*. In practice, the mycelium of *A. sinodeliciosus* can grow within the pH range of 7–8 with significant growth rate differences, while only at pH 8 does it starts to form numerous ‘hyphal knots’, which are thought of as the starting points of fruiting body formation (Fig. 4). Therefore, to further investigate the specific metabolic pathways and mechanisms which may be related to mild alkali tolerance in *A. sinodeliciosus*, we performed RNA-seq to detect the transcriptome dynamics in mycelium of *A. sinodeliciosus* under long-term neutral and mild alkali treatments. Then, functional analysis of DEGs, and gene co-expression network and key metabolic pathways analyses were conducted. Finally, based on such mechanisms and physiological processes, we also inferred the possible changes of *A. sinodeliciosus* in terms of its nutrition and flavour.

### The genomic evolution of *A. sinodeliciosus* was accompanied by whole gene family contraction, TE expansion and rapid evolution of genes

Gene family and genome duplication analyses revealed extensive gene family contraction in *A. sinodeliciosus* (Fig. 1). In particular, abundant gene families of carbohydrate hydrolases CAZymes and lignocellulosic oxidoreductases AAs and CYPs contracted, leading to *A. sinodeliciosus* having the lowest number of genes involved in depolymerization of lignin, cellulose, hemicellulose and pectin in straw rotting fungi. The fruiting body of *A. sinodeliciosus* in the field is large and fascicular, suggesting that massive gene family contraction has no effect on the growth performance of *A. sinodeliciosus*.

TE expansion in *A. sinodeliciosus* may play key roles in gene expression regulation and genome plasticity under a mild saline-alkali environment. In our study, we found an abundance of LTR-RTs amplified in *A. sinodeliciosus* since the Quaternary ice age 2 Mya (Fig. 3). Research has shown that TEs play key roles in chromosome structural variation and gene expression regulation to mediate genome adaptation and evolution [83, 84]. Therefore, identification of greatly enlarged TEs in *A. sinodeliciosus* may stimulate more in-depth studies regarding the dynamic changes of TEs and their consequences for genome evolution and function in *Agaricus*.

Rapidly evolved genes of *A. sinodeliciosus* may be involved in adaptation, including the transcription factors *seb1* and *cys-3*, and mushroom formation-related hydrophobin genes, of which *seb1* regulates gene expression, *cys-3* positively regulates sulphur-catabolism; hydrophobin is conducive to the formation of rhizomorph and primordia. For example, our previous study revealed the high sulphate concentration in the topsoil of the native habitats of *A. sinodeliciosus*, and abundant microbes associated with sulphur metabolism were found by amplicon sequencing [4], suggesting that the ability for sulphur-catabolism is necessary for organisms in their native habitats. Such findings further corroborated the rapidly evolved genes of *A. sinodeliciosus* as another genomic evolution under this particular environment.

Fungal genomes were highly remodelled during their evolution, even between closely related species [85, 86]. In rotting fungi of the genus *Agaricus*, we found this remodelling mainly involves variation in genome organization, which occurred through TE expansion, gene family contraction, whole genome duplication and rapid evolution of adaptive genes. Interestingly, these feature vary greatly among *Agaricus* that have a similar ecological niche, especially in the two narrowly distributed taxa *A. sinodeliciosus* and *A. bisporus* var. *burnettii*. *A. sinodeliciosus* can be found from the mild saline-alkali soil adjacent to deserts in northwest China [4], and *A. bisporus* var. *burnettii* is found in the Sonoran Desert of California [87]. The genome remodelling seen in *A. sinodeliciosus* during adaptive evolution involves TE expansion, gene family contraction and rapid evolution of adaptive genes, while that in *A. bisporus* var. *burnettii* involves the expansion of large families of adaptive genes and rapid evolution of adaptive genes (Fig. 2). These findings show that rotting fungi are powerful models for ecological genomics.

### Enhanced carbon and nitrogen utilization may be important advantages for *A. sinodeliciosus* grown under mild alkali conditions

Transcriptomic analysis supported that mild alkali conditions induced a significant increase of carbon and nitrogen utilization in *A. sinodeliciosus*, which involves lignocellulose degradation, lipid hydrolysis, extracellular proteolysis, sugar absorption, amino acid absorption, starch and sucrose metabolism, and biosynthesis of amino acids (Figs 5, 6 and S1). An abundance of hydrolases (CAZymes) and a small number of oxidoreductases (AAs and CYPs) were up-regulated in *A. sinodeliciosus* at pH 8.0 (Figs 5 and S1). Our previous study revealed that there was a mass of microbes involved in cellulose degradation in the native habitat of *A. sinodeliciosus* [4]. Leadbeater *et al*., using meta-exo-proteome proteomics, revealed hydrolases were the dominant lignocellulolytic enzymes in salt marsh, rather than oxidative enzymes [88]. Lipases were up-regulated in *A. sinodeliciosus* at pH 8.0 (Figs 5 and S1), which catalyse the hydrolysis of triglycerides to release free fatty acids, and enable microbes to utilize non-conventional carbon sources such as lipids [89, 90]. A series of peptidases were up-regulated in *A. sinodeliciosus* at pH 8.0 (Figs 5 and S1), which hydrolyse peptide bonds of extracellular protein to yield peptides and amino acids. Our previous study showed there was a high nitrate concentration and abundant microbes associated with nitrogen metabolism in the native habitats of *A. sinodeliciosus* [4].

Extracellular simple sugars are absorbed through major facilitator superfamily (MFS) transporters and ATP-binding cassette (ABC) transporters, and extracellular peptides and amino acids are transported into the cell by amino acid permease. These transmembrane transporters in *A. sinodeliciosus* were also up-regulated under mild alkali treatment (Figs 5 and S1). The pathways of glucose, trehalose and amino acids synthesis were significantly enhanced in *A. sinodeliciosus* after alkali treatment (Fig. 6). Glucose, as the energy substance, is involved in various primary and secondary metabolisms in organisms [91]. It has been reported that trehalose levels in plants significantly affect carbon allocation and utilization, and a higher accumulation of trehalose could result in yield improvements under abiotic stresses [92, 93]. The biosynthesis of leucine, arginine, proline, lysine, phenylalanine and tyrosine were enhanced in the pH 8.0 treatment, and *A. sinodeliciosus* also was highly effective in glutamine synthesis. Amino acids constitute the major nitrogen sources for proteins, nucleic acids, enzymes, vitamins, etc. This enhanced carbon and nitrogen acquisition and utilization may be beneficial for growth-promotion and yield improvement in *A. sinodeliciosus* under a mild saline-alkali environment. Free amino acids also have significant influence on the flavour of the mushroom, which are mainly contributors to umami taste.

### Fungal cell walls and membrane remodelling, and intracellular small molecule accumulation may be important for *A. sinodeliciosus* in tolerance to mild alkali conditions

The fungal cell wall is essential for mechanic stability during cell division and polar growth [94]. The fungal cell wall is primarily composed of chitin, and 1,3-β- and 1,6-β-glucan, so fungal chitinases (GH18) and glucanases (GH17) have a housekeeping function in cell wall remodelling and plasticity [94, 95]. It has reported that some chitinases are expressed in response to abiotic stresses in plants [96]. The enzymes GH17 and GH18 were significantly up-regulated and identified as hub genes in *A. sinodeliciosus* under mild alkali conditions (Fig. 5), which may contribute to cell division and mycelial growth, and maintain cell wall plasticity in response to this stress. Moreover, the expression of genes of lectins and hydrophobin were significantly enhanced in *A. sinodeliciosus* after alkali treatment; these are cell wall-associated proteins and are involved in the formation of aerial hyphae [97], suggesting that mild alkalinity also facilitated fruit body formation for *A. sinodeliciosus*. Numerous studies have shown that the metabolite lignin is an important strategy for plant resistance to stress [98–100], and selective lignin down-regulation leads to a constitutive defence response [101–104]. The phenylpropanoid biosynthesis pathway was inhibited in *A. sinodeliciosus* under mild alkali conditions (Fig. 6), which may contribute to increase cell wall infiltrates under mildly alkaline conditions.

It is essential to maintain the cell membrane’s stability and integrity, especially after exposure to a stress such as alkaline treatment. Lipases (triacylglycerol hydrolases) were enhanced to hydrolyse and synthesize triglycerides formed by glycerol and free fatty acids in *A. sinodeliciosus* under the mild alkali conditions, and triglycerides are essential components of the membrane. Moreover, numerous membrane proteins were up-regulated upon alkali treatment, such as pheromone A receptor, an integral membrane protein, which induces mating by stimulating a G-protein-initiated signalling pathway [105]; and transmembrane transporters (such as MFS transporters, ABC transporters, amino acid permease and ion transporters) involved in ion homeostasis, osmotic regulation and nutrient absorption.

Salinization can induce osmotic stress caused by salt and high pH, and oxidative stress by the accumulation of toxic ROS, which can damage various cell components [9, 102]. It has reported that saline-alkali-tolerant plants can regulate the biosynthesis and accumulation of small molecules, such as soluble sugars and amino acids, to improve osmotic adjustment and remove excessive ROS [106–108]. *A. sinodeliciosus* increased the absorption and biosynthesis of soluble sugars (such as glucose and trehalose) under mild alkali conditions (Fig. 6). *A. sinodeliciosus* also enhanced the absorption and biosynthesis of amino acids (including leucine, arginine, proline, lysine, phenylalanine, tyrosine and glutamine) after mild alkali exposure (Fig. 6). In addition, gene expression of the polyphenol oxidase (PPO), which mediates the biosynthesis of melanin derived from tyrosine, was enhanced in *A. sinodeliciosus* under mild alkali treatment (Fig. 6). Melanin has been reported to cause browning of button mushrooms [109], and has anti-oxidative properties [110].

### The improvement of nutrition and taste of *A. sinodeliciosus* in response to mild alkali conditions

Edible and medicinal mushrooms are nutraceutical-rich in bioactive compounds such as polysaccharides, polyphenols, proteins, lectins and terpenoids. In this study, the biosynthesis of polysaccharides (including chitin, glucose and trehalose), polyphenols (including phenylalanine and tyrosine), amino acids, lectins and terpenoids (such as sesquiterpene) were all increased under mild alkali conditions compared with neutral conditions (Fig. 6, Table S7), suggesting that mild alkali stress may contribute to the accumulation of bioactive compounds in *A. sinodeliciosus*.

In mushrooms, post-harvest lignification has been widely recognized to be a leading cause of quality deterioration in storage, because of the induction of increased toughness and leathery flesh [111]. Post-harvest lignification caused by accumulation of monolignol is mainly induced by wounding and ROS accumulation [112], and has been observed in *Pleurotus eryngii* [112], *A. bisporus* [113] and *Lentinula edodes* [114]. The phenylpropanoid biosynthesis pathway was inhibited in *A. sinodeliciosus* under mild alkali conditions (Fig. 6), suggesting that this condition may improve the quality and taste of *A. sinodeliciosus* too.

*A. sinodeliciosus* has good prospects to provide resources for meeting food needs and improving traits associated with mild saline-alkali tolerance in *Agaricus*. The variation in genomic architecture among the three closely related taxa of *Agaricus* opens a new avenue for elucidation of genome evolution under environmental stress for *Agaricus*. While using a single individual it is difficult to capture all genomic and transcriptomic variation, especially in light of physiological responses to soil environmental conditions, population genetics studies on these species would be of significance to generalize the present study from a single strain of *A. sinodeliciosus* to the species level. Furthermore, rapidly evolved genes and hub genes under mild alkali conditions in *A. sinodeliciosus* can be candidate resistance genes, to further study the molecular mechanisms under mild saline-alkali soils.

## Supplementary Data

Supplementary material 1

Supplementary material 2

Supplementary material 3

Supplementary material 4

Supplementary material 5
