# Supplementary material for: Insights into the genomic evolution and the alkali tolerance mechanisms of Agaricus sinodeliciosus by comparative genomic and transcriptomic analyses
Source: Microb Genom. 2023 Mar 8;9(3):mgen000928. doi: 10.1099/mgen.0.000928 (PMC10132060; doi:10.1099/mgen.0.000928)
Supplement: Supplementary material 5 [file mgen-9-928-s005.pdf]

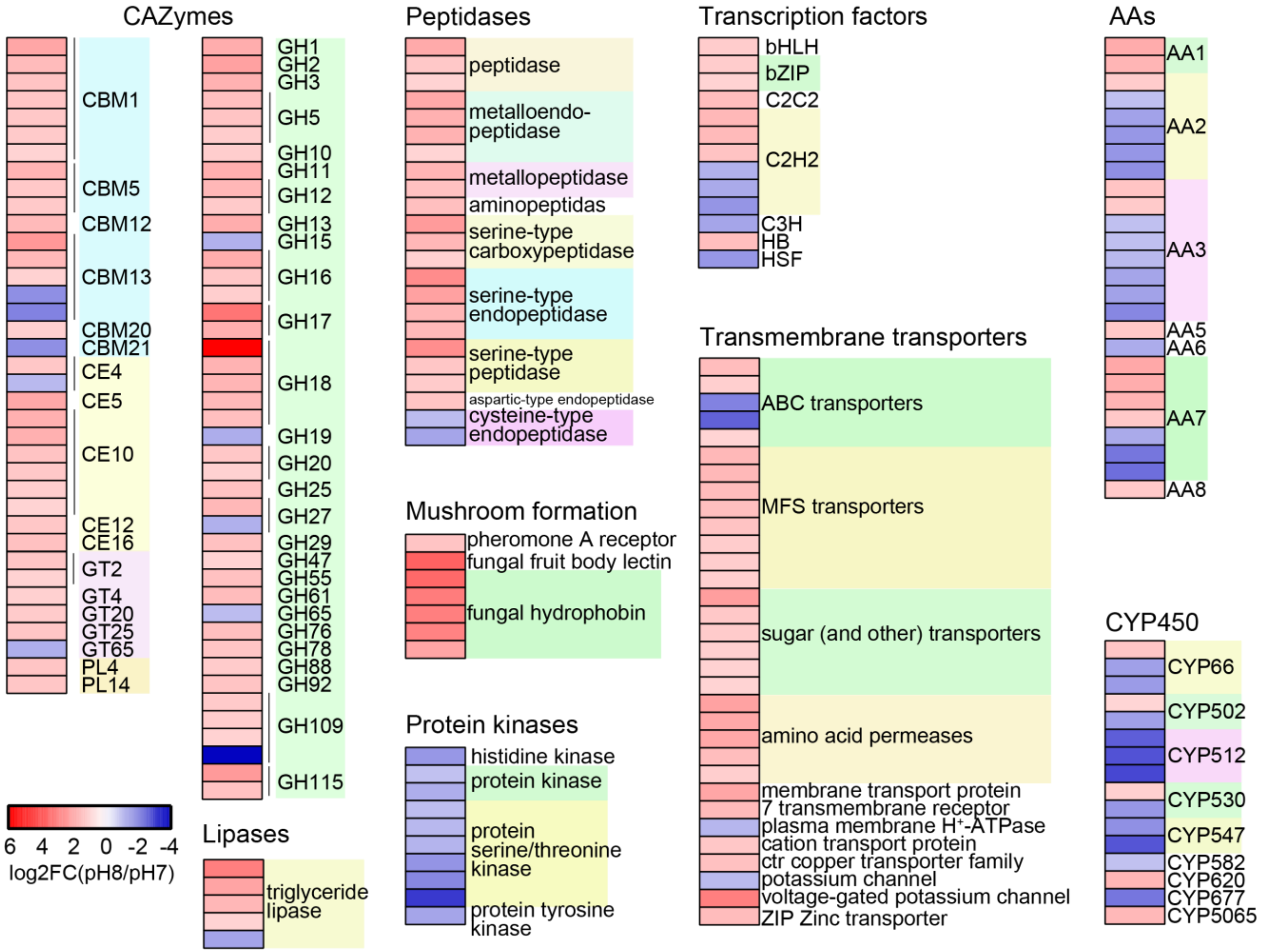

Figure S1 – Heatmap of important gene expression in differentially expressed genes (DEGs).

Table S1 The genomic information of edible and medicinal fungi used in this study.

Species	Functions	Ecological niche	Genome size (Mb)	References
Agaricus bisporus	Edible & Medicinal	straw rooting fungi	30.23	[1, 2]
Agrocybe pediades	Edible & Medicinal	straw rooting fungi	45.06	[3]
Amanita muscaria	Medicinal	symbiotic fungi	40.70	[4]
Armillaria mellea	Edible & Medicinal	white rooting fungi	79.55	[5]
Clitocybe gibba	Edible & Medicinal	straw rooting fungi	65.97	[3]
Coprinopsis cinerea	Medicinal	straw rooting fungi	37.50	[6]
Cyathus striatus	Medicinal	wood rotting fungi	91.18	[3]
Fistulina hepatica	Edible & Medicinal	brown rotting fungi	33.85	[7]
Flammulina velutipes	Edible & Medicinal	white rooting fungi	35.64	[8, 9]
Hypholoma sublateritium	Medicinal	white rooting fungi	48.03	[4]
Hypsizygus marmoreus	Edible & Medicinal	white rooting fungi	41.63	[10]
Laccaria bicolor	Edible	symbiotic fungi	60.71	[11]
Lentinula edodes	Edible & Medicinal	white rooting fungi	41.80	[12]
Lepista nuda	Edible & Medicinal	straw rooting fungi	43.49	[3]
Oudemansiella mucida	Medicinal	wood rotting fungi	61.73	[3]
Pholiota alnicola	Edible	wood rotting fungi	75.01	[3]
Pleurotus ostreatus	Edible & Medicinal	wood rotting fungi	35.60	[13-16]
Pluteus cervinus	Edible	white rooting fungi	52.19	[17]
Rhodocollybia butyracea	Edible	straw rooting fungi	96.28	[3]
Schizophyllum commune	Edible & Medicinal	white rooting fungi	38.67	[18]
Tricholoma matsutake	Edible & Medicinal	symbiotic fungi	175.76	[19]
Volvariella volvacea	Edible & Medicinal	straw rooting fungi	35.72	[20]
Scleroderma citrinum	Medicinal	symbiotic fungi	56.14	[4]
Serpula lacrymans	Medicinal	brown rotting fungi	42.73	[21]

Suillus luteus	Edible & Medicinal	symbiotic fungi	41.74	[4]
Fomitopsis pinicola	Medicinal	brown rotting fungi	41.61	[22]
Laetiporus sulphureus	Edible & Medicinal	brown rotting fungi	39.92	[23]
Pycnoporus cinnabarinus	Medicinal	white rooting fungi	35.14	[24, 25]
Trametes versicolor	Medicinal	white rooting fungi	44.79	[22]
Wolfiporia cocos	Edible & Medicinal	brown rotting fungi	50.48	[22]
Stereum hirsutum	Medicinal	white rooting fungi	46.51	[22]
Neolentinus lepideus	Edible	brown rotting fungi	35.64	[23]
Gloeophyllum trabeum	Medicinal	brown rotting fungi	37.18	[22]
Punctularia strigosozonata	NA	white rooting fungi	34.17	[26]
Phanerochaete chrysosporium	NA	white rooting fungi	35.15	[27]
Fomitiporia mediterranea	NA	white rooting fungi	63.35	[26]
Auricularia subglabra	Edible	white rooting fungi	76.85	[26]
Calocera cornea	Edible	wood rotting fungi	33.24	[23]
Calocera viscosa	Edible	wood rotting fungi	29.10	[23]
Tremella mesenterica	Edible & Medicinal	symbiotic fungi	28.64	[22]
Ustilago maydis	NA	pathogen	19.66	[28]
Aspergillus niger	Outgroup	pathogen	34.85	[29]

NA: not announced.

References:

1. **Morin E, Kohler A, Baker AR, Foulongne-Oriol M, Lombard V *et al***. Genome sequence of the button mushroom Agaricus bisporus reveals mechanisms governing adaptation to a humic-rich ecological niche. *Proceedings of the National Academy of Sciences of the United States of America* 2012; 109:17501-17506.
2. **Sonnenberg ASM, Sedaghat-Telgerd N, Lavrijssen B, Ohm RA, Hendrickx PM *et al***. Telomere-to-telomere assembled and centromere annotated genomes of the two main subspecies of the button mushroom *Agaricus bisporus* reveal especially polymorphic chromosome ends. *Sci Rep* 2020; 10:14653.
3. **Ruiz-Dueñas FJ, Barrasa JM, Sánchez-García M, Camarero S, Miyauchi S *et al***. Genomic analysis enlightens Agaricales lifestyle evolution and increasing peroxidase diversity.

Mol Biol Evol 2021; 38:1428-1446.

4. **Kohler A, Kuo A, Nagy LG, Morin E, Barry KW *et al***. Convergent losses of decay mechanisms and rapid turnover of symbiosis genes in mycorrhizal mutualists. *Nat Genet* 2015; 47:410-415.
5. **Collins C, Keane TM, Turner DJ, O'Keeffe G, Fitzpatrick DA *et al***. Genomic and proteomic dissection of the ubiquitous plant pathogen, *Armillaria mellea*: toward a new infection model system. *J Proteome Res* 2013; 12:2552-2570.
6. **Stajich JE, Wilke SK, Ahren D, Au CH, Birren BW *et al***. Insights into evolution of multicellular fungi from the assembled chromosomes of the mushroom Coprinopsis cinerea (Coprinus cinereus). *Proceedings of the National Academy of Sciences of the United States of America* 2010; 107:11889-11894.
7. **Floudas D, Held BW, Riley R, Nagy LG, Koehler G *et al***. Evolution of novel wood decay mechanisms in Agaricales revealed by the genome sequences of Fistulina hepatica and Cylindrobasidium torrendii. *Fungal genetics and biology : FG & B* 2015; 76:78-92.
8. **Park YJ, Baek JH, Lee S, Kim C, Rhee H *et al***. Whole genome and global gene expression analyses of the model mushroom *Flammulina velutipes* reveal a high capacity for lignocellulose degradation. *PLoS ONE* 2014; 9:e93560.
9. **Yoon H, You YH, Woo JR, Park YJ, Kong WS *et al***. The mitochondrial genome of the white-rot fungus Flammulina velutipes. *Journal of General & Applied Microbiology* 2012; 58:331-337.
10. **Zhang J, Ren A, Chen H, Zhao M, Shi L *et al***. Transcriptome analysis and its application in identifying genes associated with fruiting body development in basidiomycete *Hypsizygus marmoreus*. *PLoS ONE* 2015; 10:e0123025.
11. **Martin F, Aerts A, Ahren D, Brun A, Danchin EG *et al***. The genome of *Laccaria bicolor* provides insights into mycorrhizal symbiosis. *Nature* 2008; 452:88-92.
12. **Chen L, Gong Y, Cai Y, Wei L, Yan Z *et al***. Genome Sequence of the Edible Cultivated MushroomLentinula edodes(Shiitake) Reveals Insights into Lignocellulose Degradation. *PloS one* 2016; 11.
13. **Riley R, Salamov AA, Brown DW, Nagy LG, Floudas D *et al***. Extensive sampling of basidiomycete genomes demonstrates inadequacy of the white-rot/brown-rot paradigm for wood decay fungi. *Proceedings of the National Academy of Sciences of the United States of America* 2014; 111:9923-9928.
14. **Alfaro M, Castanera R, Lavin JL, Grigoriev IV, Oguiza JA *et al***. Comparative and transcriptional analysis of the predicted secretome in the lignocellulose-degrading basidiomycete fungus Pleurotus ostreatus. *Environmental microbiology* 2016; 18:4710-4726.
15. **Ramirez L, Oguiza JA, Perez G, Lavin JL, Omarini A *et al***. Genomics and transcriptomics characterization of genes expressed during postharvest at 4 degrees C by the edible basidiomycete Pleurotus ostreatus. *International microbiology : the official journal of the Spanish Society for Microbiology* 2011; 14:111-120.
16. **Castanera R, Lopez-Varas L, Borgognone A, LaButti K, Lapidus A *et al***. Transposable Elements versus the Fungal Genome: Impact on Whole-Genome Architecture and Transcriptional Profiles. *PLoS genetics* 2016; 12:e1006108.

17. **Varga T, Krizsán K, Földi C, Dima B, Sánchez-García M *et al***. Megaphylogeny resolves global patterns of mushroom evolution. *Nat Ecol Evol* 2019; 3:668-678.
18. **Ohm RA, de Jong JF, Lugones LG, Aerts A, Kothe E *et al***. Genome sequence of the model mushroom Schizophyllum commune. *Nat Biotechnol* 2010; 28:957-963.
19. **Miyauchi S, Kiss E, Kuo A, Drula E, Kohler A *et al***. Large-scale genome sequencing of mycorrhizal fungi provides insights into the early evolution of symbiotic traits. *Nat Commun* 2020; 11:5125.
20. **Bao D, Gong M, Zheng H, Chen M, Zhang L *et al***. Sequencing and comparative analysis of the straw mushroom (*Volvariella volvacea*) genome. *PLoS ONE* 2013; 8:e58294-e58294.
21. **C ED, D F, M B, Majcherczyk A, Schneider P *et al***. The Plant Cell Wall–Decomposing Machinery Underlies the Functional Diversity of Forest Fungi. *Science* 2011; 333:762-765.
22. **D F, M B, R R, Barry K, Blanchette RA *et al***. The Paleozoic Origin of Enzymatic Lignin Decomposition Reconstructed from 31 Fungal Genomes. *Science* 2012; 336:1715-1719.
23. **Nagy LG, Riley R, Tritt A, Adam C, Daum C *et al***. Comparative genomics of early-diverging mushroom-forming fungi provides insights into the origins of lignocellulose decay capabilities. *Mol Biol Evol* 2016; 33:959-970.
24. **Levasseur A, Lomascolo A, Chabrol O, Ruiz-Duenas FJ, Boukhris-Uzan E *et al***. The genome of the white-rot fungus *Pycnoporus cinnabarinus*: a basidiomycete model with a versatile arsenal for lignocellulosic biomass breakdown. *BMC Genomics* 2014; 15:486.
25. **Busk PK, Lange M, Pilgaard B, Lange L**. Several genes encoding enzymes with the same activity are necessary for aerobic fungal degradation of cellulose in nature. *PloS one* 2014; 9:e114138.
26. **Floudas D, Binder M, Riley R, Barry K, Blanchette RA *et al***. The paleozoic origin of enzymatic lignin decomposition reconstructed from 31 fungal genomes. *Science* 2012; 336:1715-1719.
27. **Ohm RA, Riley R, Salamov A, Min B, Choi IG *et al***. Genomics of wood-degrading fungi. *Fungal Genet Biol* 2014; 72:82-90. 10.1016/j.fgb.2014.05.001.
28. **Kämper J, Kahmann R, Bölker M, Ma L-J, Brefort T *et al***. Insights from the genome of the biotrophic fungal plant pathogen *Ustilago maydis*. *Nature* 2006; 444:97-101.
29. **Andersen MR, Salazar MP, Schaap PJ, van de Vondervoort PJ, Culley D *et al***. Comparative genomics of citric-acid-producing *Aspergillus niger* ATCC 1015 versus enzyme-producing CBS 513.88. *Genome Res* 2011; 21:885-897.

Table S2 Secondary metabolite gene clusters of *A. sinodeliciosus*.

**Cluster code**	**Location**	**Gene cluster type**	**Genome region**	**Gene number in gene cluster**
Cluster 1	Contig28	terpene	1,682,935 - 1,704,858	10
Cluster 2	Contig28	NRPS-like	1,931,912 - 1,977,451	16
Cluster 3	Contig10	NRPS-like	707,038 - 750,554	14
Cluster 4	Contig36	T1PKS	2,024,984 - 2,071,042	19
Cluster 5	Contig40	terpene	241,543 - 263,024	7
Cluster 6	Contig51	NRPS-like	171,520 - 215,641	8
Cluster 7	Contig54	indole	122,645 - 144,114	3
Cluster 8	Contig54	terpene	204,267 - 225,931	4
Cluster 9	Contig5	NRPS-like	3,346,072 - 3,373,510	6
Cluster 10	Contig5	siderophore	3,498,751 - 3,514,370	4
Cluster 11	Contig5	terpene	3,700,741 - 3,718,800	8
Cluster 12	Contig5	terpene	4,436,524 - 4,458,283	7
Cluster 13	Contig5	NRPS-like,T1PKS	4,617,842 - 4,676,824	16
Cluster 14	Contig14	terpene	32,159 - 44,836	4
Cluster 15	Contig14	terpene	1,827,740 - 1,848,912	11
Cluster 16	Contig16	NRPS-like	111,608 - 155,146	17
Cluster 17	Contig16	terpene	529,099 - 550,361	7

Table S5 Genome duplications of four closely related taxa.

**Type of duplication**	***A. sinodeliciosus***	***A. bisporus* var. *bisporus***	***A. bisporus* var. *burnettii***	***C. cinerea***
Singleton	4348	4491	4560	5592
Dispersed duplicated genes	3610	3719	3889	4966
Proximal duplicated genes	526	682	724	980
Tandem duplicated genes	539	878	993	1156
WGD or segmental duplicated genes	63	675	1597	699
**Total genes**	9086	10445	11763	13393
**percentage of duplicated genes (%)**	**52.15**	**57**	**61.23**	**58.25**
